# Influence of Primary Palatal Surgery on Craniofacial Morphology in Patients with Cleft Palate Only (CPO)—Systematic Review with Meta-Analysis

**DOI:** 10.3390/ijerph192114006

**Published:** 2022-10-27

**Authors:** Joanna Janiszewska-Olszowska, Katarzyna Grocholewicz, Marta Mazur, Maciej Jedliński

**Affiliations:** 1 Department of Interdisciplinary Dentistry, Pomeranian Medical University in Szczecin, 70-111 Szczecin, Poland; 2 Department of Oral and Maxillofacial Sciences, Sapienza University of Rome, 00161 Rome, Italy

**Keywords:** cleft palate, CPO, craniofacial malformations, craniofacial morphology, palatal surgery, SNA angle, cephalometry, cephalometric analysis

## Abstract

Background: Cleft palate only (CPO) is the second most prevalent cleft type. Both the cleft and palatal scarring may affect craniofacial growth. The aim of this systematic review was to summarize scientific evidence on effect of palatal surgery on craniofacial morphology in CPO. Methods: A search was conducted in PubMed, PMC, WoS, Scopus, Embase, using the keywords: “cleft palate” AND (“craniofacial morphology” OR “cephalometric analysis”) NOT “lip” with inclusion and exclusion criteria ensuring confident, direct comparison between study groups. The quality assessment was performed with Arrive’s scale for radiologic examinations. Results: Of 713 potential articles, 19 were subjected to qualitative analysis and 17 to meta-analysis, which confirmed reduced SNA in unoperated CPO versus non-cleft individuals. No scientific evidence was found directly assessing the effect of surgery on craniofacial morphology. The negative effect of palatal surgery was seen indirectly: in treated CPO versus non-cleft, the size effect of SNA is bigger than in untreated CPO versus non-cleft. A high heterogeneity came from a few non-European publications. Conclusions: CPO is associated with sagittal maxillary deficiency resulting both from the cleft and from primary surgery, disregarding cleft severity in operated CPO patients. Ethnic differences influence craniofacial morphology in CPO. This research received no external funding. Study protocol number in PROSPERO database: CRD42021268957.

## 1. Introduction

Cleft palate only (CPO) is the second most prevalent type of cleft (0.1–1.1 per 1000 births) with the sex ratio opposite to that for cleft lip and palate (CLP), (male/female = 0.90) [1]. It affects eating and chewing, speech, occlusion, and facial appearance, e.g., features of high importance for every person. Thus, cleft palate repair is aimed at improving the social and psychological well-being of the patients affected.

Referring to craniofacial growth, it is evident, that patients with clefts are characterized by impaired maxillary growth. It may be supposed that craniofacial growth is affected both by the cleft itself and by tissue scarring from palatal surgery. On the other hand, it is difficult to identify evidence referring to craniofacial morphology in CPO, since the terms “cleft palate” and “isolated cleft palate” are used in the literature for different types of clefts.

Both CLP and CPO may have a family history with several family members affected. However, CLP and CPO rarely occur together within the same family [2]. Thus, it is considered that relatives of individuals with CLP are at risk of CLP, but not of CPO [2]. It is assumed that patients with CLP, and those with CPO, should not be grouped together in scientific studies due to differences in etiology and morphology. The design of scientific studies on patients affected by clefts should separately analyze groups of patients with clefts involving the lip and palate from those with the palate involved only. Nevertheless, many studies can be found comparing patients with various types of clefts, without non-cleft control groups of the same ethnicity [3,4,5,6,7].

The aim of the present systematic review was to find and summarize existing scientific evidence concerning the effect of palatal surgery on the growth and development of craniofacial structures in patients with cleft of the palate without cleft lip.

## 2. Materials and Methods

### 2.1. Search Strategy

**Figure 1:**
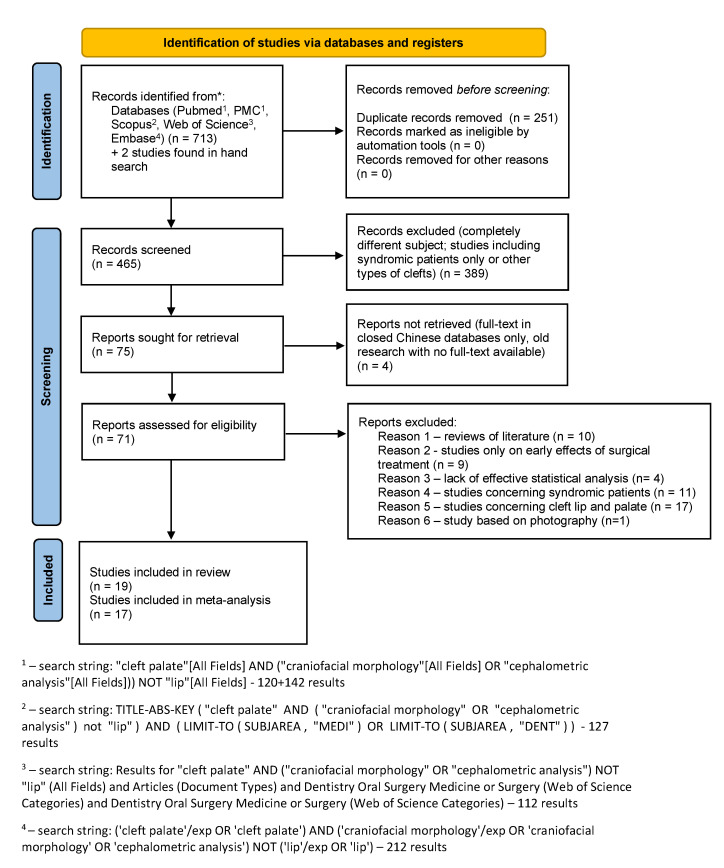
Prisma 2020 flow diagram.

This systematic review was conducted according to the PRISMA statement [8], the PRISMA reporting guidelines [9,10] [Supplementary Materials S1 and S2] and the guidelines from the Cochrane Handbook for Systematic Reviews of Interventions [11]. In accordance with PICO [12], the framework of the present systematic review is as follows: Population: patients with non-syndromic cleft palate only; Intervention: primary palatal surgery; Comparison: operated versus non-operated patients with non-syndromic cleft palate only; non-cleft healthy population; Outcomes: lateral cephalometric measurements. The PICO question was the following: “Does the craniofacial morphology of cleft palate only untreated patients differ to this of non-cleft healthy population? Does the craniofacial morphology of operated cleft palate only patients differ to non-cleft healthy population? Does the craniofacial morphology of operated cleft palate only patients differ to untreated ones? Does the extent of cleft palate only influence the craniofacial morphology?” On 14 February 2021 a series of pre-searches of the following databases was performed: PubMed, PubMed Central, Web of Science, Scopus, Embase. Then, the study protocol was registered in PROSPERO database (Ref. No CRD42021268957) on 10 September 2021. Subsequently, the final search proceeded on 1 December 2021 and was updated on 16 May 2022 in the following databases: PubMed, PubMed Central, Web of Science, Scopus, Embase, using the following keywords: “cleft palate” AND (“craniofacial morphology” OR “cephalometric analysis”) NOT “lip”. A hand search was performed in reference lists of the papers included. The exact search string for each database used is described on PRISMA 2020 flow diagram (Figure 1).

### 2.2. Eligibility Criteria

For the present systematic review, the following inclusion criteria were applied:

Type of study: observational studies, cohort studies, case-control studies, retrospective studies on craniofacial morphology of patients with cleft palate only.
Outcome of interest: hard and soft tissue craniofacial morphology in lateral cephalometric analysis.

Object of the study: (a) comparison of craniofacial morphology in unoperated cleft palate only patients to non-cleft healthy population and (b) comparison of craniofacial morphology in operated CPO patients to non-cleft healthy population and (c) effect of cleft palate extent on craniofacial morphology.

Subject of the study: human subjects

The exclusion criteria were as follows: studies not referring to CPO, studies not using cephalometric analysis, animal studies, case reports, reviews, lack of effective statistical analysis, studies referring to effect of surgical procedures other than palatal closure (e.g., distraction osteogenesis, LeFort osteotomies, pharyngeal flap surgery, mandibular set-back osteotomy and other), studies based on infant or prenatal craniofacial measurements, studies on patients with diagnosed syndromes, studies including cleft lip and palate (CLP) patients, studies on non-cleft family members of patients with cleft palate. 

### 2.3. Data Extraction

After retrieving the results from the search engines to create a database, the duplicates were removed. Then, titles and abstracts were analyzed by two authors independently (MJ and KG), following the inclusion criteria. Subsequently, the full text of each selected article was then analyzed to verify whether it was suitable for inclusion and exclusion criteria. Whenever a disagreement occurred, it was resolved by a discussion with the third author (JJO) by creating a working spreadsheet in order to compare them in accordance with the Cochrane collaboration guidelines [4]. Data were sought regarding the changes within the craniofacial morphology, studied with cephalometric analysis. No methods of cephalometric analysis were excluded. The authors were extracting cephalometric values from papers included, in order to find ones that were used in most of the studies and thus could be compared. The Cohen’s K coefficient for the agreement between the authors in study selection indicates a high agreement between the authors as was equal to 0.978. Authorship, year of publication, type of each eligible study and its main results regarding to the craniofacial morphology of CPO patients were extracted by one author (JJO) and examined by another author (MJ).

### 2.4. Quality Assessment

According to the PRISMA statements, an evaluation of methodological quality was performed in order to properly evaluate the strength of evidence provided by the included studies, as methodological flaws can result in biases [8].

In the case of studies based on the observation of structures in radiological examinations, a specific scale for Clinical Studies of Radiologic Examinations could be applied. In the case of the present study, it was decided to use an Arrive’ scale for radiologic examinations [13]. It consists of the following factors: study design, study purpose, reference standard, inclusion criteria, indeterminate results, exclusion criteria, spectrum of patients, analysis method, analysis criteria, avoided work-up bias, avoided diagnostic-review bias, avoided test-review bias, intraobserver reliability, interobserver reliability, and statistical analysis. The abovementioned structure allowed us to assess accurately the risk of bias, and due to its complexity, provided a detailed analysis of the results. One point was given for the compliance of the characteristics with the requirements listed in the scale. In the event of a defect in the methodology, the following study receives 0 points. The higher score the studies receives, the better the morphology in which it is characterized.

The quality assessment was performed independently by two authors (JJO and MJ). In case of quality assessment, the Cohen’s K coefficient for the agreement between the authors was 0.99. Whenever a disagreement occurred, it was resolved by another author (MM).

### 2.5. Meta-Analysis

Meta-analysis was performed with the R statistical program, Version 4.1.2 [14] using random-effect model via metafor R package [15], with Mean Differences (MD) and 95% confidence intervals (95% CI) being calculated as effect estimates. Heterogeneity was assessed quantitatively using I2-statistics and Cochran’s Q [16]. The results were considered statistically significant at *p* < 0.05. Publication bias was estimated using a funnel plot.

## 3. Results

### 3.1. Results of the Search

The search strategy identified 713 potential articles: 120 from PubMed, 142 from PubMed Central, 127 from Scopus, 112 from Web of science and 221 from Embase. Additionally, two papers were added following the hand search. After 251 duplicates had been removed, 465 articles were analyzed. Subsequently, 389 papers were excluded because they did not meet the inclusion criteria. Of the remaining 75 papers, 37 were excluded because they were not relevant to the subject of the study (as they were characterized as reviews of literature, studies on early effects of surgical treatment, lack of effective statistical analysis, studies concerning syndromic patients, and studies concerning cleft lip and palate). Thus, finally 19 papers were subjected to a qualitative analysis and 17 to a meta-analysis. The whole process is described on Prisma 2020 Flow Diagram (Figure 1. Flow diagram) The main characteristics of each included study are presented in Table 1.

### 3.2. Quality Assesment

The Results of the assesment are presented in Table 2.

The overall quality of the evidence is high- or medium-quality; none of the included studies was characterized by a low quality. Error study (re-examination of the lateral cephalograms or reassessment by another clinician) and power study were not performed in all the studies. Most of the studies did not address the limitations that might arise from the design of a study. This is due to the good patient selection and a common and widely recognized examination method (cephalometric analysis).

### 3.3. Meta-Analysis

The following meta-analysis was performed in order to compare the range of changes within craniofacial morphology. For this purpose, values such as the age of the patients under study and the SNA and ANB angles were used. If such a value was not provided, the study was excluded from the meta-analysis. Extracted data which served as the basis for the meta-analysis are presented in Table 3. In order to get a broad picture of the craniofacial morphology of the patient population with CPO, five different comparisons presented below were performed.

#### 3.3.1. Patients with Untreated Cleft Palate Only vs. Non-Cleft Healthy Population

##### SNA

**Figure 2:**
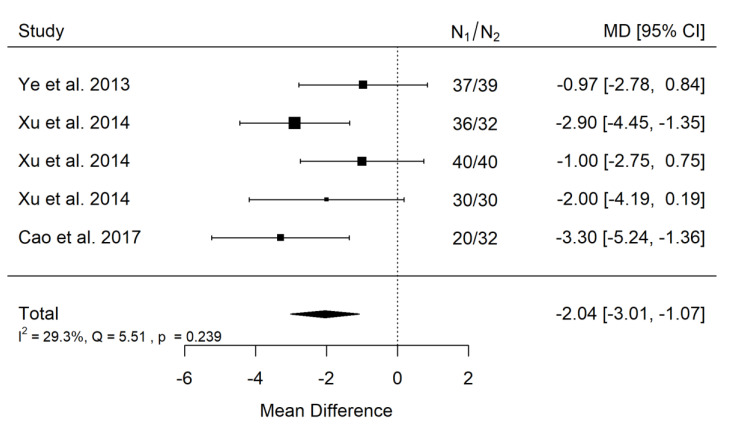
Forest plot of SNA values for the comparison of patients with untreated cleft palate only vs. non-cleft healthy population [18,34,35].

**Figure 3:**
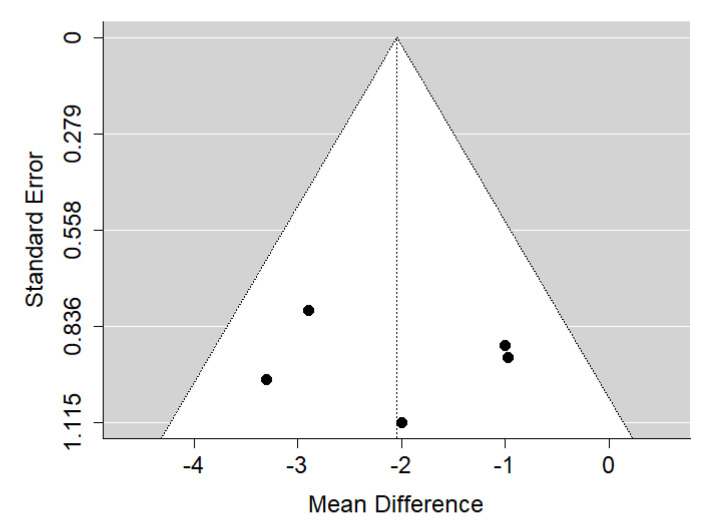
Funnel plot of SNA values for the comparison of patients with untreated cleft palate only vs. non-cleft healthy population [18,34,35].

There is very large significant (*p* < 0.001) negative effect size. Study results were consistent, heterogeneity was insignificant (*p* = 0.239), and only about 29% of the variability came from heterogeneity (Figure 2). The funnel plot did not reveal publication bias (Figure 3). Patients with untreated cleft palate only were characterized by lower values of SNA angle than a healthy population of the same ethnic origin.

##### ANB

**Figure 4:**
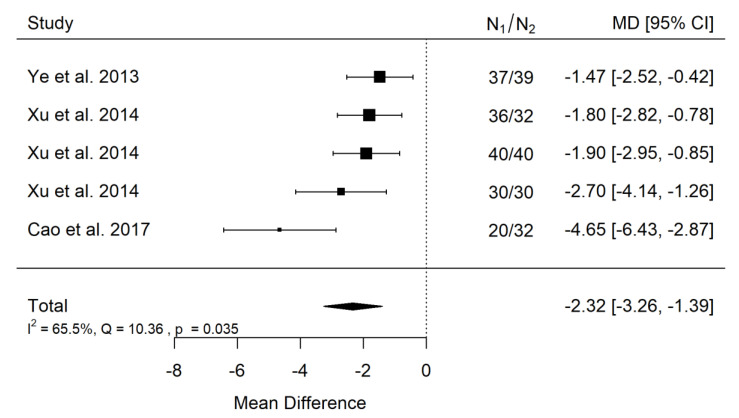
Forest plot of ANB values for the comparison of patients with untreated cleft palate only vs. non-cleft healthy population [18,34,35].

**Figure 5:**
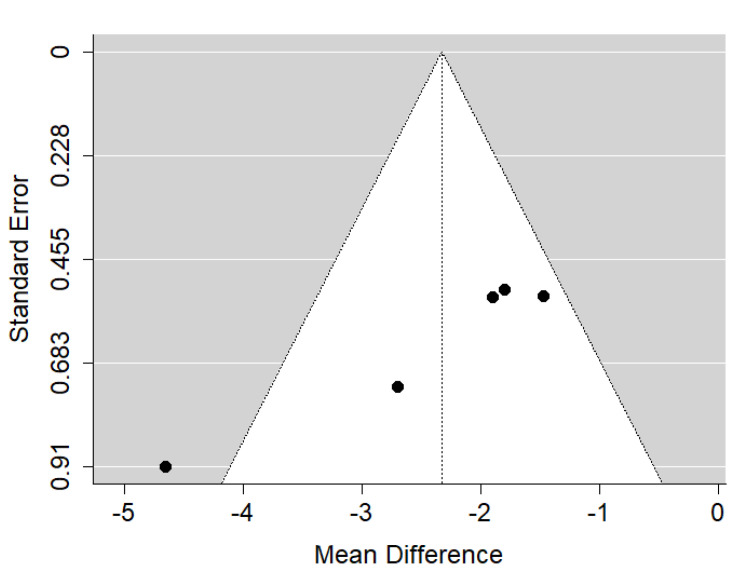
Funnel plot of ANB values for the comparison of patients with untreated cleft palate only vs. non-cleft healthy population [18,34,35].

There is a very large significant (*p* < 0.001) negative effect size. Study results were inconsistent and heterogeneity was significant (*p* = 0.035), about 66% of the variability came from heterogeneity (Figure 4). The funnel plot suggests some publication bias, coming from the study of Cao et al. (Figure 5). However, due to the value of heterogeneity, no binding conclusion can be issued.

#### 3.3.2. Patients with Untreated Submucous Cleft Palate vs. Non-Cleft Healthy Population

##### SNA

**Figure 6:**
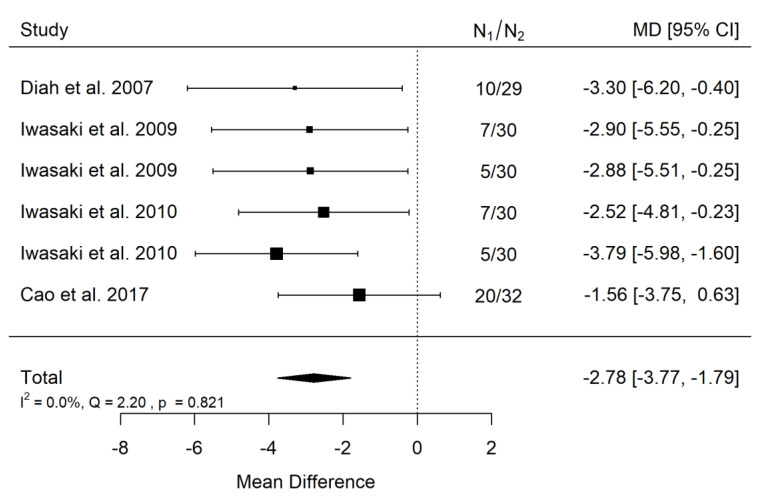
Forest plot of SNA values for the comparison of patients with untreated submucous cleft palate vs. non-cleft healthy population [18,21,24,25].

**Figure 7:**
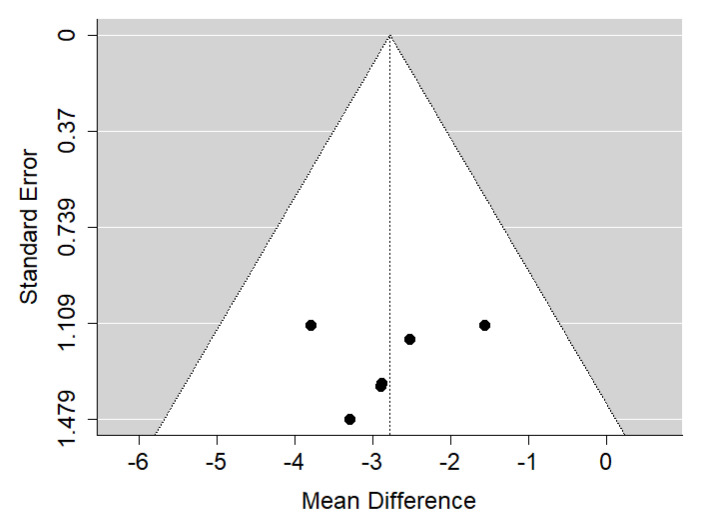
Funnel plot of SNA values for the comparison of patients with untreated submucous cleft palate vs. non-cleft healthy population [18,21,24,25].

There is very large significant (*p* < 0.001) negative effect size. Study results were consistent, heterogeneity was insignificant (*p* = 0.821), almost no variability came from heterogeneity (Figure 6). The funnel plot did not reveal publication bias (Figure 7). Patients with untreated submucous cleft palate are characterized by lower values of SNA angle than a healthy population of the same ethnic origin.

##### ANB

**Figure 8:**
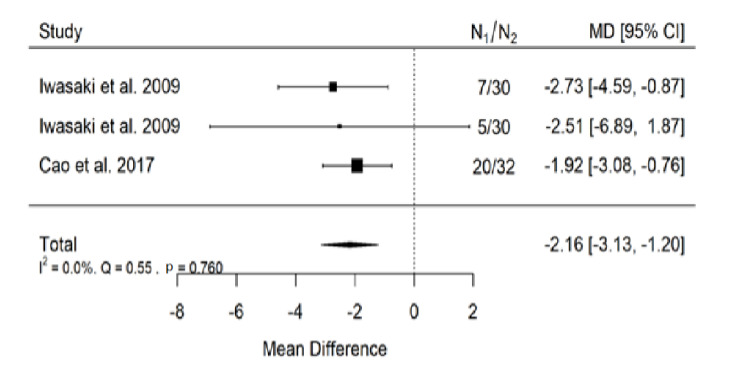
Forest plot of ANB values for the comparison of patients with untreated submucous cleft palate vs. non-cleft healthy population [18,24].

**Figure 9:**
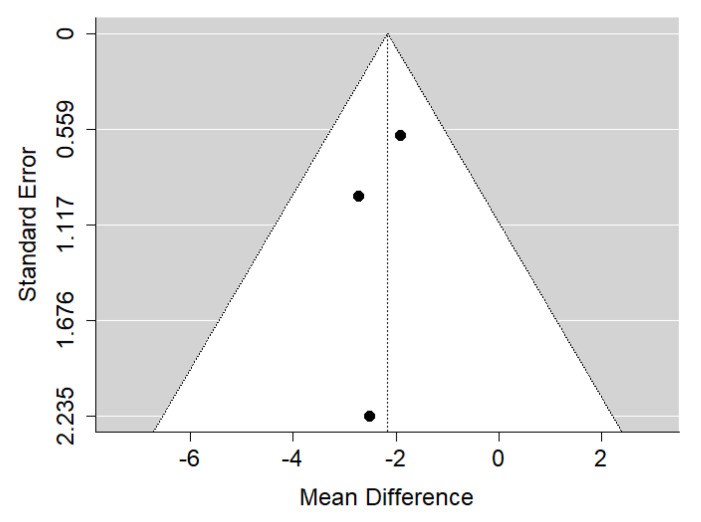
Funnel plot of ANB values for the comparison of patients with untreated submucous cleft palate vs. non-cleft healthy population [18,24].

There is very large significant (*p* < 0.001) negative effect size. Study results were consistent, heterogeneity was insignificant (*p* = 0.760), almost no variability came from heterogeneity (Figure 8). The funnel plot did not reveal publication bias (Figure 9). Patients with untreated submucous cleft palate are characterized by lower values of ANB angle than a healthy population of the same ethnic origin.

#### 3.3.3. Patients with Cleft Palate Only Treated Surgically and Orthodontically vs. Non-Cleft Healthy Population

##### SNA

**Figure 10:**
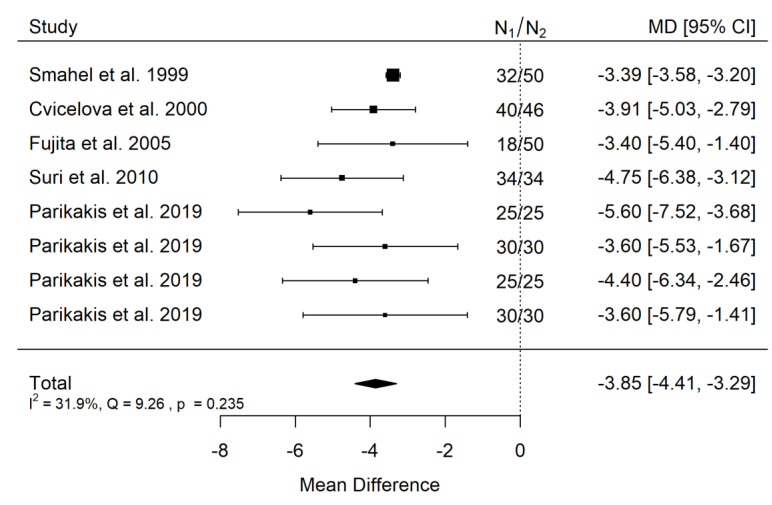
Forest plot of SNA values for the comparison of patients with cleft palate only treated surgically and orthodontically vs. non-cleft healthy population [19,22,29,32,33].

**Figure 11:**
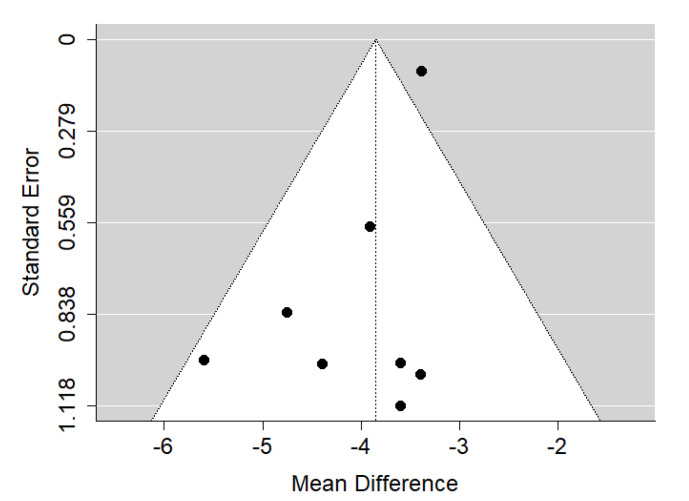
Funnel plot of SNA values for the comparison of patients with cleft palate only treated surgically and orthodontically vs. non-cleft healthy population [19,22,29,32,33].

There is very large significant (*p* < 0.001) negative effect size. Study results were consistent, heterogeneity was insignificant (*p* = 0.235), about 32% of the variability came from heterogeneity (Figure 10). The funnel plot did not suggest evident publication bias (Figure 11). The heterogeneity may come from different surgical and orthodontic techniques applied in every single study. However, the funnel plot did not suggest a publication bias. This means that patients with CPO treated surgically and orthodontically have much more lower values of SNA that non-cleft healthy population of the same ethnic origin. It should be noted here that their negative effect size is bigger than that of the untreated CPO to non-cleft healthy population.

##### ANB

**Figure 12:**
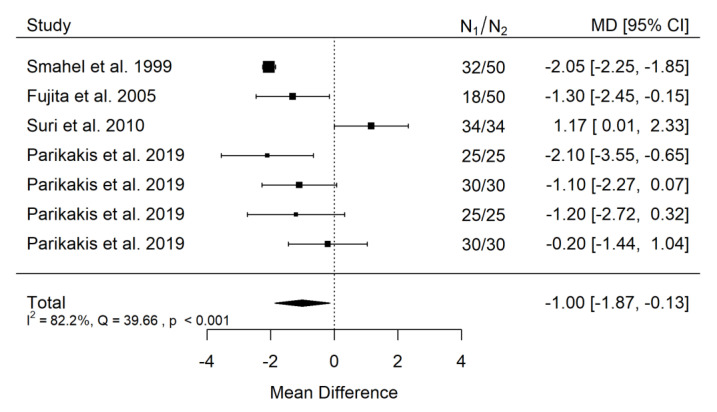
Forest plot of ANB values for the comparison of patients with cleft palate only treated surgically and orthodontically vs. non-cleft healthy population [19,22,29,32,33].

**Figure 13:**
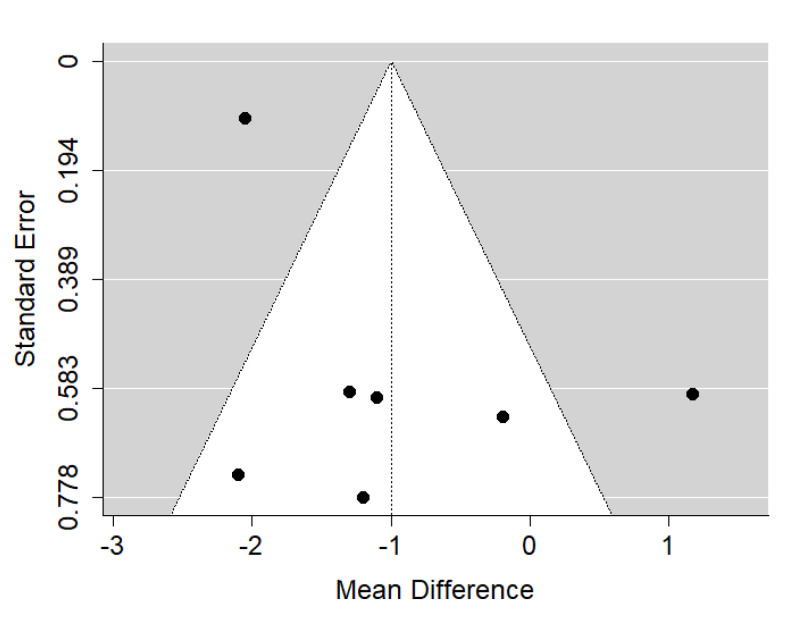
Funnel plot of ANB values for the comparison of patients with cleft palate only treated surgically and orthodontically vs. non-cleft healthy population [19,22,29,32,33].

There is large significant (*p* = 0.024) negative effect size. Study results were inconsistent, and heterogeneity was significant (*p* < 0.001), about 82% of the variability came from heterogeneity (Figure 12). The funnel plot asymmetry may suggest some publication bias (Figure 13). 

Also in this point, is must be underlined, that in such a situation, in which subjects slightly differ from each other between the groups, such heterogeneity cannot be avoided. It should be noted here that their negative effect size is smaller than that of the untreated CPO to non-cleft healthy population.

#### 3.3.4. Patients with Submucous Cleft Palate Treated Surgically and Orthodontically vs. Non-Cleft Healthy Population

##### SNA

**Figure 14:**
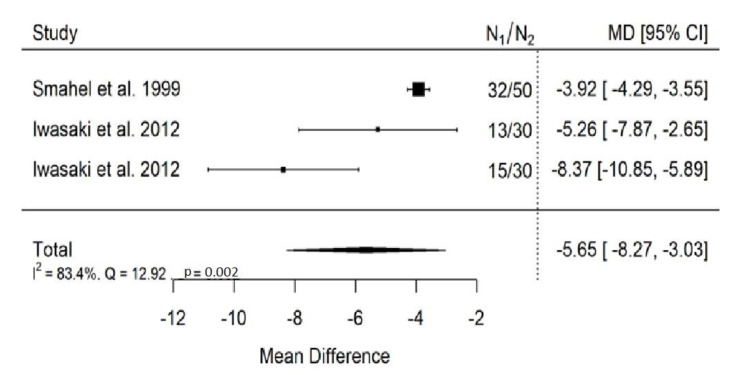
Forest plot of SNA values for the comparison of patients with submucous cleft palate treated surgically and orthodontically vs. non-cleft healthy population [26,32].

**Figure 15:**
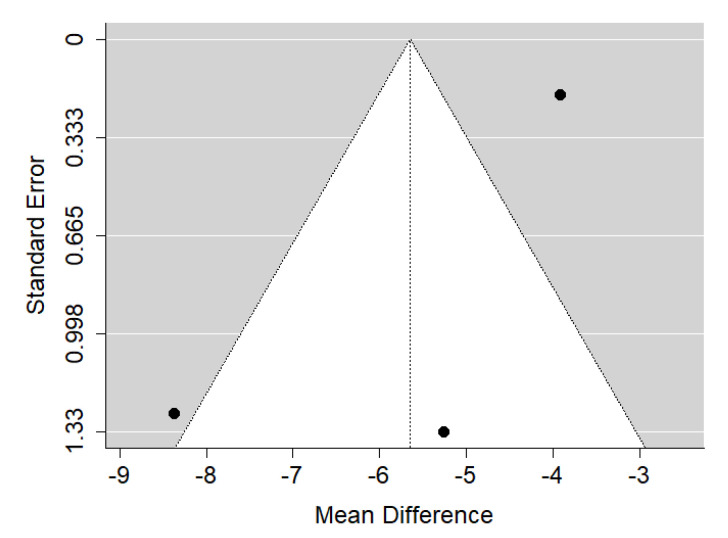
Funnel plot of SNA values for the comparison of patients with submucous cleft palate treated surgically and orthodontically vs. non-cleft healthy population [26,32].

There is very large significant (*p* < 0.001) negative effect size. Study results were inconsistent and heterogeneity was significant (*p* = 0.002), about 83% of the variability came from heterogeneity (Figure 14). The funnel plot suggests some publication bias (Figure 15).

##### ANB

Only one study reported ANB value—no meta-analysis could be performed.

#### 3.3.5. The Influence of Severity of Cleft Palate on the Following Skeletal Changes of Face over the Course of Life of Treated Patients

##### SNA

**Figure 16:**
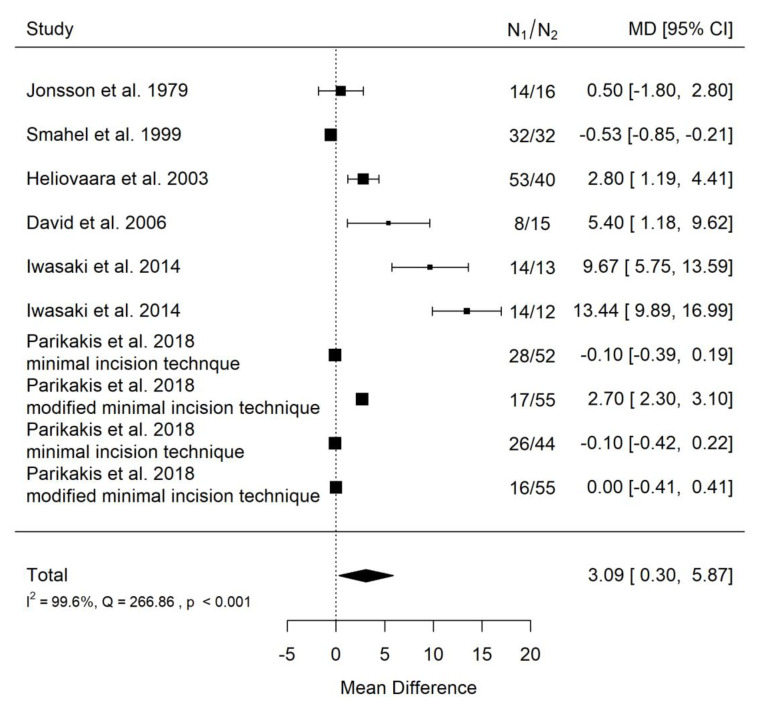
Forest plot of SNA values for the comparison of CPO patients of different age [20,23,27,28,30,32].

**Figure 17:**
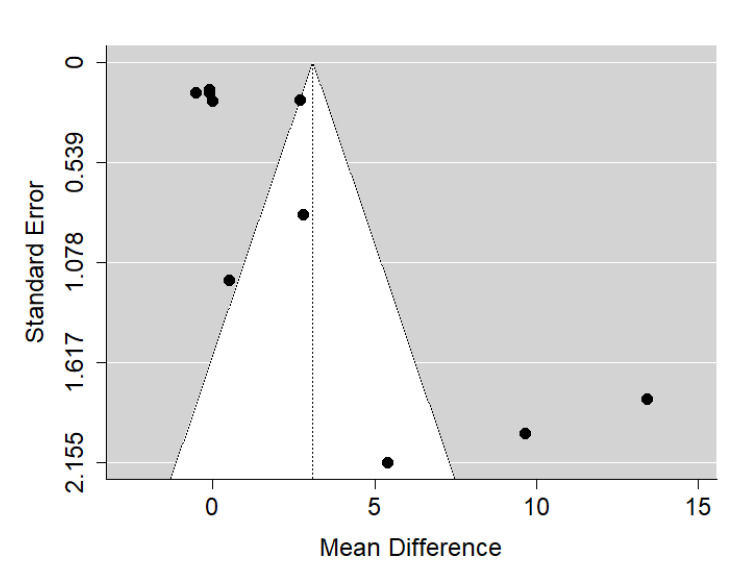
Funnel plot of SNA values for the comparison of CPO patients of different age [20,23,27,28,30,32].

There is very large significant (*p* = 0.030) positive effect size. Study results were very inconsistent, and heterogeneity was significant (*p* < 0.001), almost all the variability come from heterogeneity (Figure 16). The funnel plot asymmetry suggests a publication bias (Figure 17). However, such extreme heterogeneity came solely from publications by David et al. and Iwasaki et al. Probably, without these publications, effect size would be insignificant. This means that studies conducted on European populations only are more consistent and show no influence of cleft palate severity on the craniofacial morphology of treated patients.

##### ANB

**Figure 18:**
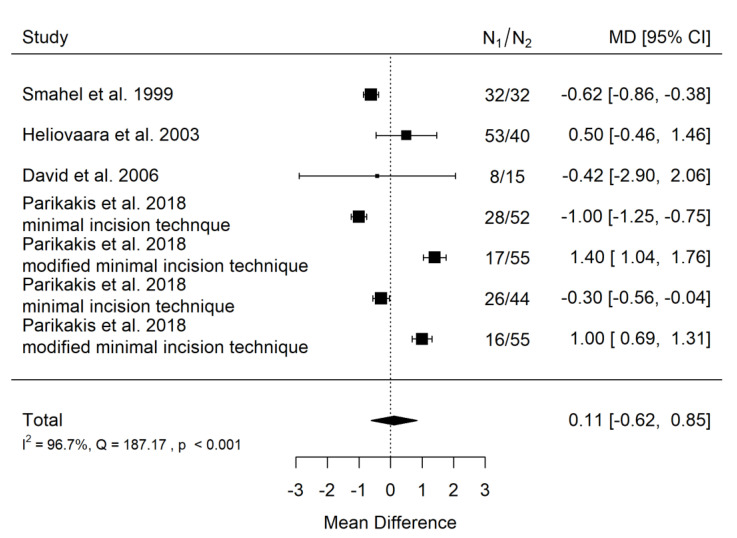
Forest plot of SNA values for the comparison of CPO patients of different age [20,23,30,32].

**Figure 19:**
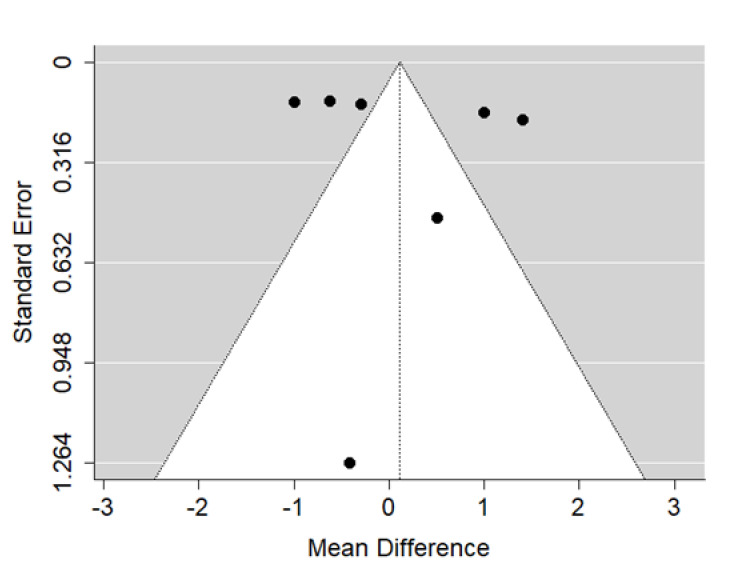
Funnel plot of ANB values for the comparison of CPO patients of different age [20,23,30,32].

There is very small and insignificant (*p* = 0.763) positive effect size. Study results were very inconsistent, and heterogeneity was significant (*p* < 0.001), about 97% of the variability came from heterogeneity (Figure 18). Most points on the funnel plot are outside the funnel due to high heterogeneity, the funnel plot did not suggest a publication bias (Figure 19).

## 4. Discussion

Unfortunately, the influence of palatal surgery in CPO on craniofacial growth cannot be directly assessed, since no studies have been found comparing unoperated versus operated CPO patients referring to craniofacial morphology.

The fact that patients with untreated CPO are characterized by lower SNA values than a healthy population of the same ethnicity indicates a negative effect of CPO on maxillary anterior growth (very large significant negative effect size). Referring to ANB (which is a difference between SNA and SNB) in the unoperated CPO patients versus non-cleft individuals, the fact that study results are inconsistent (about 66% of the variability come from heterogeneity) is caused by a more severe sagittal discrepancy in patients included in the paper by Cao et al. [18]. This seems to be caused both by a more severe maxillary deficiency (than in other papers) as well as probably by larger mandibles. The fact that the effect size is large indicates that CPO results in sagittal skeletal discrepancy.

In the comparison between patients who have undergone surgical and orthodontic treatment and non-cleft individuals, the size effect on SNA was much bigger than in untreated CPO individuals compared to non-cleft patients, possibly indirectly indicating a further negative effect of palatal surgery in CPO on the sagittal jaw relationship of the patients affected. In contrary, the effect is not visible for ANB angle because of a high heterogeneity and a funnel plot suggesting publication bias.

As far as submucous cleft palate is concerned, diagnostic criteria including bifid uvula, translucent midline zone, and an absent bony palate posterior border were proposed by Calnan in 1954. Patients with submucous cleft palate may be characterized by a different severity of velopharyngeal disfunction, not always indicating a need for a surgical intervention. It is evident that maxillary deficiency is caused by the cleft itself since patients with untreated submucous cleft palates are characterized by lower values of SNA angle than a healthy population of the same ethnic origin. Patients with operated sCPO are characterized by a maxillary deficiency; they have significantly lower SNA than healthy controls. This effect is present in different populations and age ranges. Thus, growth retardation could be related both to the cleft itself and to the surgery. Unfortunately, no meta-analysis of the ANB angle could be made between patients with unoperated sCPO and healthy controls, since not enough data could be found in the literature.

The fact that a comparison between operated sCPO and operated CPO patients revealed a limited influence of cleft severity on SNA and ANB in operated CPO patients, may confirm the negative impact of palatal surgery on maxillary sagittal growth. Moreover, the high heterogeneity came from a limited number of non-European publications. This fact indicates that ethnic differences are of importance when analyzing craniofacial morphology.

It was difficult to select relevant studies due to a diversity of the terminology used. The search terms had to exclude the word “lip” since a high variety of names are used for a cleft posterior to incisal foramen. The most precise term seems “cleft palate only”, however it is not widely used. The term “isolated cleft palate” is by some authors used as a synonym for “nonsyndromic cleft” [36,37] and by some as “cleft palate without cleft lip” [38] or as any type of cleft involving the palate [5] or for cleft lip and palate.

Patients with different syndromes (Apert, Crouzon, Pfeiffer, cleidocranial dysplasia, Saethre–Chotzen, Stickler and others) may have craniofacial morphology characteristics for each syndrome, being the subjects of few papers—thus studies on patients with diagnosed syndromes were excluded from the review in order to obtain uniformity. Robin sequence was not considered an exclusion criterion. Robin sequence is not a syndrome, but rather a series of symptoms and has no strict uniform diagnostic criteria.

The cephalometric variables used are different among the papers included. Thus, the authors of the present study have decided to use SNA and ANB, which were reported in most papers included. Using the angles, SNA describing sagittal maxillary position and ANB—sagittal intermaxillary relationship made it necessary to limit the quantitative comparisons to the sagittal configuration of the maxilla. Unfortunately, no uniform cephalometric analysis exists, and there is no consensus on describing cephalometric craniofacial morphology. Moreover, it should be remembered that not all morphological characteristics of the craniofacial skeleton are all visible in lateral cephalometric radiographs.

It should be underlined that the findings of the present meta-analysis, referring to maxillary sagittal morphology, may not be consistent to studies using different variables to assess the maxilla. Thus, contrary conclusions referring to influence of cleft severity (CPO versus SCPO) are drawn by Cao et al. [18], who used maxillary length described by the values of ANS-PMP, A-PMP and Ba-ANS and found a statistically significant difference between the study groups.

A limitation of the present study comes from the fact that only sagittal variables could be found in most studies and used for quantitative assessment. It is evident that vertical cephalometric craniofacial morphology in CPO patients differs from a healthy population as well: Iwasaki et al. [26], report a lower posterior facial height in operated versus unoperated patients with sCPO, which may result from palatal surgery and the resulting tissue scarring. However, diverse use of various cephalomertric measurements makes it impossible to perform a meta-analysis.

## 5. Conclusions

1. Unoperated CPO patients are characterized by a sagittal maxillary deficiency compared to non-cleft individuals;
2. No direct scientific evidence could be found allowing for the direct assessment of the influence of palatal surgery in CPO on craniofacial morphology;
3. A negative effect of palatal surgery in CPO on the sagittal jaw relationship in the patients affected can be indirectly seen in the following comparisons: in patients who have undergone surgical and orthodontic treatment comparing to non-cleft individuals, the size effect of SNA angle is bigger than in untreated CPO individuals compared to non-cleft;
4. Cleft severity has a limited influence on SNA and ANB in operated CPO patients;
5. Ethnic differences seems to be of importance when analyzing craniofacial morphology in CPO.

## Figures and Tables

**Table 1:** Characteristics of included studies.

Author and Year	Study Groups		Outcome Measured	Results
Caillot et al., 2017 [17]	patients with Pierre-Robin sequence at age 6 years operated at mean age 6 months (*n* = 15)	patients with Pierre-Robin sequence at age 6 years operated at ages 12–18 months (*n* = 10)	Lateral cephalometric analysis	SNA and SNB angles present smaller values in patients operated at earlier ages. ANB angle was similar between the groups.
Cao et al., 2017 [18]	unoperated adults with submucous cleft palate (sCPO) (*n* = 20) and unoperated CPO adult patients (*n* = 20)	non-cleft controls aged 18–30 years (*n* = 32)	Lateral cephalometric analysis	Sagittal maxillary length (ANS-PMP, A-PMP and Ba-ANS) in CPO patients was smaller than in SCPO and in sCPO was smaller than in non-cleft group.
Cvicelova et al. [19]	operated CPO aged 7–9 years old (*n* = 40)	non-cleft controls aged 7–9 years (*n* = 46)	Lateral cephalometric analysis	SNA (angle F) presents smaller values in CPO patients. Mandibular angle (angle G) was significantly lower. Mandibule was significantly retracted in respect to maxilla and cranial base.
David et al., 2006 [20]	Patients with sCPO (*n* = 8) aged 15–18 years old	Patients with non-syndromic CPO aged 16–20 years old (*n* = 15)	Lateral cephalometric analysis and audiometry	Cephalometric analysis at skeletal maturity revealed a reduced SNA angle in CPO compared to sCPO. Hearing was within the defined normal limits for all but two patients. One patient was severely impaired on one side (–40 dB) and required a hearing aid, and the other patient also had a unilateral loss; in this case, –30 dB. Speech results were judged to be within normal limits, many still had mild articulation errors. Resonance was similarly very common at some stage during development in both male and female patients, with 28 of the 32 patients affected.
Diah et al., 2007 [21]	Patients with unoperated CPO (*n* = 10)	Non-cleft healthy individuals (*n* = 29)	Lateral cephalometric analysis and 3D digital models analysis	SNA value is significantly lower in CPO patients. Additionally, their palatal surface is much smaller
Fujita et al., 2005 [22]	Patients after prepubertal growth spurt (mean 17.6 years) with operated CPO (*n* = 18)	Non-cleft healthy controls after prepubertal growth spurt (mean 17.6 years) (*n* = 34)	Lateral cephalometric analysis	In CPO patients maxillary length was shorter and the nasomaxillary complex was positioned more posteriorly in relation to the anterior cranial base, compared with the controls. The craniofacial pattern in the CPO patients was characterized as a bimaxillary retrusion, a more counterclockwise rotation of the mandible, and a shorter mandible than in non-cleft subjects.
Heliovaara et al., 2003 [23]	CPO girls aged 6 years (*n* = 60)	CPO girls aged 6 years (*n* = 53)	Lateral cephalometric analysis	Both maxilla and mandible were retruded, sCPO patients had a higher degree of maxillary and mandibular soft tissue prominence masking the skeletal retrusion.
Iwasaki 2009 [24]	children with unoperated sCPO at age 9 (*n* = 12)	non-cleft children with normal occlusion aged 9 (*n* = 60)	Lateral cephalometric analysis	Maxillary length was reduced in sCPO children compared to unaffected individuals.
Iwasaki et al., 2010 [25]	patients with sCPO aged 14 (*n* = 12)	non-cleft children with normal occlusion aged 14 (*n* = 60)	Lateral cephalometric analysis	In sCPO patients maxillary length was shorter, anterior part of the maxilla was retruded and posterior part of the maxilla was in a more anterior position, the inclination of the palatal plane was more pronounced compared to non-cleft children
Iwasaki et al., 2012 [26]	Patients with sCPO: operated at mean ages 3.7 years at 9 and 14 years old (*n* = 28)	unoperated patients, examined at ages 9 and 14 (*n* = 13)	Lateral cephalometric analysis	Posterior facial height was significantly shorter and palatal plane was more inclined in operated patients. Posterior part of the maxilla was positioned more posteriorly in operated versus unoperated patients.
Iwasaki et al., 2014 [27]	patients with sCPO (*n* = 12), CPO patients not extending as far as incisive foramen (*n* = 13), patients reaching to incisive foramen (*n* = 12); all age-matched		Lateral cephalometric analysis	In children with CPO anteroposterior maxillary length was shorter, and anterior part of the maxilla was positioned more posteriorly.
Jonsson et al., 1979 [28]	patients with sCPO (*n* = 26)	CPO patients (*n* = 29)	Lateral cephalometric analysis	CPO children had lower SNA values, higher gonial angle and higher posterior face height.
Parikakis et al., 2019 [29]	CPO patients operated at age 13 months with cephalograms made at ages 10 and 16 years (*n* = 55)	non-cleft children with 55 cephalograms made at age 10 years and 55 at age of 16 years, results presented for boys and girls separately (*n* = 110/2)	Lateral cephalometric analysis	SNA and SNB were smaller, palatal plane and mandibular length were shorter and posterior upper face height was shorter in CPO patients compared to non-cleft individuals.
Parikakis et al., 2018 [30]	170 Caucasian operated patients with CPO The patients were treated surgically with minimal-incicsion (*n* = 85) or minial-incision with muscle reconstruction (*n* = 85) palatoplasty and divided further into two subgroups: clefts within the soft palate only (*n* = 51) and within the hard and soft palate (*n* = 119)		Lateral cephalometric analysis	At 5 years of age, an increased inclination of the palatal plane to anterior cranial base, decreased posterior upper face height, and a shorter mandibular length were found in the CPO group. At 10 years of age, an increased inclination of the palatal plane, a decreased posterior upper face height, and a longer palatal length were found in the minimal-incision group with muscle reconstruction.
Parikakis et al., 2018 [31]	145 children with non-syndromic CPO: with sCPO (*n* = 34) and with soft and hard palate repaired with Veau-Wardill-Kilner technique (*n* = 25)	children with sCPO (*n* = 30), children with CPO repaired (*n* = 56) with minimal incision technique	Lateral cephalometry at ages 5 and 10	Mandibular length at age 5 was shorter in minimal incision group. ANB angle was smaller in Veau-Wardill-Kilner technique group. No significant differences were found at age 10.
Smahel et al. 1999 [32]	187 adult men patients with different types of cleft palate including patients with sCPO (*n* = 17) and CPO patients (*n* = 32)		Lateral cephalometrics	SNA was lower, inclination of upper incisors to nasion-pogonion line was lower, proportion of posterior to anterior face height was lower than in uncleft controls.
Suri et al. 2021 [33]	34 patientes with operated non-syndormic cleft palate (*n* = 34)	Non-cleft healthy controls (*n* = 34)	Lateral cephalometrics	Significant differences were noted in CPO group regarding smaller cranial base length, shorter maxillary length, increased palatal and mandibular plane inclinations.
Xu et al., 2014 [34]	Unoperated Chineese CPO patients (*n* = 106)	Non-cleft healthy controls (*n* = 102)	Anteroposterior and lateral cephalometrics	Unoperated children showed a shorter cranial base length (S-N, S-Ba, N-Ba), a reduced maxillary horizontal length (ANS-Pmp), reduced maxillary vertical dimension (N-ANS) and retruded maxilla (SNA). The mandibular body, ramus and total mandibular length were shortened. However, sagittal mandibular position (SNB) and chin (Sn-Pg) were not significantly different from unaffected controls. Maxillary transverse dimensions were normal.
Ye et al. 2013 [35]	Nonsyndromic isolated CPO (*n* = 37)	Age and gender matched non-clefts (*n* = 39)	Lateral cephalometrics	Patients with isolated cleft palate were characterized by maxillary retrusion. Mandible morphology and cranial basal morphology in CPO showed no significant difference with controls. Patients with CPO are more vulnerable to cross bite. Intrinsic deficiencies did detrimental effect on maxilla sagittal length, but did no detrimental effect on maxilla position, mandible size and position.

**Table 2:** Characteristics—according to Arrive’ scale for radiologic examinations [13].

Authors and Year of Publication	Study Design	Study Purpose	Reference Standard	Inclusion Criteria	Indeterminate Results	Exclusion Criteria	Spectrum of Patients	Analysis Method	Analysis Criteria	Avoided Work-Up Bias	Avoided Diagnostic-Review Bias	Avoided Test-Review Bias	Intraobserver Reliability	Interobserver Reliability	Statistical Analysis
Caillot et al., 2018 [17]	1	1	1	1	1	1	1	1	1	1	1	1	1	0	1
Cao et al., 2017 [18]	1	1	1	1	1	1	1	1	1	1	1	0	1	0	1
Cvicelova et al. [19]	1	1	1	1	0	0	1	1	1	1	0	0	0	0	1
David et al., 2006 [20]	1	1	1	1	0	1	1	1	1	1	1	1	0	0	1
Diah et al., 2007 [21]	1	1	1	1	1	1	1	1	1	1	1	1	1	0	1
Fujita et al., 2005 [22]	1	1	1	1	1	1	1	1	1	1	1	1	1	0	1
Heliovaara et al., 2003 [23]	1	1	1	1	1	0	1	1	1	0	0	0	0	0	1
Iwasaki 2009 [24]	1	1	1	1	1	1	1	1	1	0	0	0	0	0	1
Iwasaki et al. 2010 [25]	1	1	1	1	1	1	1	1	1	0	0	0	0	0	1
Iwasaki et al., 2012 [26]	1	1	1	1	1	1	1	1	1	0	0	0	0	0	1
Iwasaki et al., 2014 [27]	1	1	1	1	0	1	0	1	1	0	0	0	0	0	1
Jonsson et al., 1979 [28]	1	1	1	1	1	0	0	1	1	1	0	0	0	0	1
Parikakis et al., 2019 [29]	1	1	1	1	1	1	1	1	1	1	1	1	1	0	1
Parikakis et al., 2018 [30]	1	1	1	1	1	1	1	1	1	1	1	1	1	0	1
Parikakis et al., 2018 [31]	1	1	1	1	1	1	1	1	1	1	1	1	1	0	1
Smahel et al., 1999 [32]	1	1	1	1	1	0	1	1	1	1	0	0	0	0	1
Suri et al., 2021 [33]	1	1	1	1	1	1	1	1	1	1	1	1	1	0	1

**Table 3:** Extracted data subjected to meta-analysis.

Patients with Untreated Cleft Palate Only vs. Non-Cleft Healthy Population								
Author	Number of Cleft Patients	Age of Cleft Patients in Years	SNA Value in the Cleft Group	ANB Value in the Clef Group	Number of Healthy Patients	Age of Healthy Patients in Years	SNA Value in the Healthy Group	ANB Value in the Healthy Group
Cao et al., 2017 [18]	20	25.43 ± 7.18	78.24 ± 3.55	−0.71 ± 3.9	32	24.65 ± 6.16	81.54 ± 3.34	3.94 ± 1.48
Xu et al., 2014 [34]	36	5–7	77.5 ± 3.3	2.4 ± 2.3	32	5–7	80.4 ± 3.2	4.2 ± 2.0
Xu et al., 2014 [34]	40	12–14	80.2 ± 4.5	1.1 ± 3.0	40	12–14	81.2 ± 3.4	3.0 ± 1.6
Xu et al., 2014 [34]	30	>18	79.4 ± 4.7	0.8 ± 3.6	30	>18	81.4 ± 3.9	3.5 ± 1.8
Ye et al., 2013 [35]	37	22.19 ± 6.57	79.30 ± 4.39	0.51 ± 2.22	39	21.31 ± 5.27	80.27 ± 3.62	1.98 ± 2.45
Patients with untreated submucous cleft palate vs. non-cleft healthy population								
Author	Number of cleft patients	Age of clef ± patients in years	SNA value in the cleft group	ANB value in the clef group	Number of healthy patients	Age of healthy patients in years	SNA value in the healthy group	ANB value in the healthy group
Cao et al., 2017 [18]	20	24.32 ± 6.22	79.98 ± 4.23	2.02 ± 2.38	32	24.65 ± 6.16	81.54 ± 3.34	3.94 ± 1.48
Diah et al., 2007 [21]	10	>16	80.8 ± 4.4	Not provided	29	>18	84.1 ± 2.7	Not provided
Iwasaki et al., 2009 [24]	7	9.5 ± 0.4	80.35 ± 3.12	1.91 ± 2.22	30	9.5 ± 0.3	83.25 ± 3.60	4.64 ± 2.43
Iwasaki et al., 2009 [24]	5	9.5 ± 0.4	79.99 ± 2.55	1.82 ± 4.91	30	9.5 ± 0.4	82.87 ± 3.87	4.33 ± 2.28
Iwasaki et al., 2010 [25]	7	14 (14.1–14.9)	85.40 ± 2.50	Not provided	30	14 (14.1–14.9)	87.92 ± 3.78	Not provided
Iwasaki et al., 2010 [25]	5	14 (14.1–14.9)	83.71 ± 1.84	Not provided	30	14 (14.1–14.9)	87.50 ± 4.14	Not provided
Patients with cleft palate only treated surgically and orthodontically vs. non-cleft healthy population								
Author	Number of cleft patients	Age of cleft patients in years	SNA value in the cleft group	ANB value in the clef group	Number of healthy patients	Age of healthy patients in years	SNA value in the healthy group	ANB value in the healthy group
Cvicelova et al., 2000 [19]	40	7–9	80.14 ± 2.06	Not provided	46	7–9	84.05 ± 3.18	Not provided
Fujita et al., 2005 [22]	18	Mean: 17 years 6 months; range: 14 years 7 months to 22 years 6 months	78.6 ± 4.33	1.8 ± 2.18	50	>18	82 ± 0.06	3.1 ± 2.03
Parikakis et al., 2019 [29]	25	Mean 10	77.9 ± 3.2	2 ± 3.1	25	Mean 10	83.5 ± 3.7	4.1 ± 2
Parikakis et al., 2019 [29]	30	Mean 10	80.4 ± 4.2	2.9 ± 2.6	30	Mean 10	84.0 ± 3.4	4 ± 2
Parikakis et al., 2019 [29]	25	Mean 16	79.6 ± 3.5	1.02.8	25	Mean 16	84.0 ± 3.5	2.2 ± 2.7
Parikakis et al., 2019 [29]	30	Mean 16	81.1 ± 4.8	2.32.9	30	Mean 16	84.7 ± 3.8	2.5 ± 1.9
Smahel et al., 1999 [32]	32	20–40	77.45 ± 0.08	0.59 ± 0.51	50	20–40	80.84 ± 0.68	2.64 ± 0.35
Suri et al., 2010 [33]	34	11 ± 0.7	76.53 ± 3.75	4.39 ± 2.97	34	11.9 ± 0.9	81.28 ± 3.09	3.22 ± 1.78
Patients with submucous cleft palate treated surgically and orthodontically vs. non-cleft healthy population								
Author	Number of cleft patients	Age of cleft patients	SNA value in the cleft group	ANB value in the clef group	Number of healthy patients	Age of healthy patients in years	SNA value in the healthy group	ANB value in the healthy group
Iwasaki et al., 2012 [26]	13	14 (14.1–14.9)	83.2 ± 4.3	Not provided	30	14 (14.1–14.9)	88.46 ± 3.23	Not provided
Iwasaki et al., 2012 [26]	15	14 (14.1–14.9)	80.19 ± 3.96	Not provided	30	14 (14.1–14.9)	88.56 ± 4.1	Not provided
Smahel et al., 1999 [32]	32	20–40	76.92 ± 0.92	−0.03 ± 0.47	50	20–40	80.84 ± 0.68	2.64 ± 0.35
The influence of severity of cleft palate on the following skeletal changes of face over the course of life of treated patients								
Author	Number of patients with submucosal cleft palate	Age of patients with submucosal cleft palate in years	SNA value in patients with submucosal cleft palate	ANB value in patients with submucosal cleft palate	Number of patients with cleft palate only	Age of patients with cleft palate only in years	SNA value in patients with cleft palate only	ANB value in patients with cleft palate only
David et al., 2006 [20]	8	16.375 ± 0.99	83 ± 5.39	1.38 ± 2.82	15	17.3 ± 1.07	77.6 ± 3.9	1.8 ± 3.01
Heliovaara et al., 2003 [23]	53	Mean: 6.2 (5.5–7.5)	92.2 ± 4.2	8.3 ± 2.4	40	Mean: 6.2 (5.8–6.8)	89.4 ± 3.7	7.8 ± 2.3
Iwasaki et al., 2014 [27]	14	Mean: 9.4 (9.0–9.9 years)	85.57 ± 5.47	Not provided	13—not extending to incisive foramen	Mean: 9.4 (9.0–9.9 years)	75.90 ± 4.92	Not provided
Iwasaki et al., 2014 [27]	14	Mean: 9.4 (9.0–9.9 years)	85.57 ± 5.47	Not provided	12—extending to incisive foramen	Mean: 9.4 (9.0–9.9 years)	72.13 ± 3.71	Not provided
Jonsson et al., 1979 [28]	14	10	77.8 ± 3.2	Not provided	16	10	77.3 ± 3.2	Not provided
Smahel et al., 1999 [32]	32	20–40	76.92 ± 0.92	−0.03 ± 0.47	32	20–40	77.45 ± 0.08	0.59 ± 0.51
Parikakis et al., 2018—minimal incision technique [30]	28	5	80.7 ± 0.7	3.8 ± 0.6	52	5	80.8 ± 0.5	4.8 ± 0.4
Parikakis et al., 2018—modified minimal incision technique [30]	17	5	82.8 ± 0.8	4.9 ± 0.7	55	5	80.1 ± 0.5	3.5 ± 0.5
Parikakis et al., 2018—minimal incision technique [30]	26	10	80.2 ± 0.7	2.9 ± 0.6	44	10	80.3 ± 0.6	3.2 ± 0.4
Parikakis et al., 2018—modified minimal incision technique [30]	16	10	80.1 ± 0.8	3.3 ± 0.6	55	10	80.1 ± 0.5	2.3 ± 0.4

N1/N2 are numbers of patients in the left/right part of the table. Negative values of mean difference mean smaller angles in patients in the left part of the Table 3.

## Data Availability

Not applicable.

## Supplementary Materials

The following are available online at https://www.mdpi.com/article/10.3390/ijerph192114006/s1, S1: PRISMA 2020 abstract checklist, S2: PRISMA 2020 checklist.
